# When Patient Rudeness Impacts Care: A Review of Incivility in Healthcare

**DOI:** 10.7759/cureus.40521

**Published:** 2023-06-16

**Authors:** Alexandra Townsley, Jennifer Li-Wang, Rajani Katta

**Affiliations:** 1 Psychology, Rice University, Houston, USA; 2 English, Rice University, Houston, USA; 3 Internal Medicine, Baylor College of Medicine, Houston, USA; 4 Dermatology, University of Texas Health Science Center at Houston, Houston, USA

**Keywords:** healthcare worker well-being, mistreatment, patient incivility, rude behavior, rudeness, incivility

## Abstract

Healthcare workers increasingly face incivility and rude behaviors from patients, families, and visitors. Although these are less severe than other types of mistreatment, studies have documented that they may still impact healthcare worker well-being and patient care. Defining and measuring incivility can be challenging because current research relies on the perceptions of the targets. Furthermore, there is often overlap among different types of mistreatment, and much of it goes unreported by those who experience it. Nevertheless, multiple studies have documented that incivility is common in healthcare and has been associated with burnout and intent to leave. In clinical settings, multiple consequences for patient care have been documented, including adverse consequences in the diagnostic and intervention performance of teams, as well as team processes. One theory is that incivility incidents divert cognitive resources away from the intervention and that these experiences may interfere with higher-order reasoning. Although limited research has been performed in the areas of prevention, response to incidents of incivility, and best practices for ameliorating the effects of incivility, some promising interventions have been reported in the literature.

## Introduction and background

One perception in the modern world is that rude behavior is on the rise. Researchers who study rudeness and incivility faced by frontline workers in multiple fields have documented that rates are indeed rising. Dr. Christine Porath, an incivility researcher, has surveyed workers globally. She reports that in 2005, nearly half of workers stated they were treated rudely at work at least once a month. In 2011, this rose to 55%, and in 2016, to 62%. In 2022, she surveyed more than 2,000 frontline workers in more than 25 industries and found that 76% reported experiencing incivility at least once a month [1].

These challenges are certainly faced by healthcare workers. During the pandemic, healthcare workers faced numerous challenges. In some cases, this included mistreatment from patients, families, and visitors. One survey study completed during the pandemic found that physicians commonly experienced mistreatment, with the most frequent source being patients and visitors. Mistreatment was associated with increased occupational distress, including increases in burnout and intent to leave the profession as well as decreases in professional fulfillment [2].

Mistreatment covers a spectrum of negative behaviors. In this review, we specifically focus on rudeness and incivility. These behaviors, though less severe than other types of mistreatment, may still result in significant impacts on healthcare worker well-being and patient care. On the more severe end of the mistreatment spectrum lie discrimination, sexual harassment, and workplace violence, subjects that are not covered in this review. 

In this narrative review of incivility directed at healthcare workers from patients, families, and visitors, we focus on defining and measuring these behaviors, as well as their impact on the workforce and healthcare. We also review documented strategies to prevent incivility and/or mitigate its effects.

## Review

Defining and measuring incivility

In order to determine the true prevalence and impact of incivility, it is important to define it clearly and measure it accurately. It is also important to identify the source of these negative interactions. Cash et al. report that in a pool of 2,815 EMS workers, 47.4% experienced incivility from some source at least once per week [3]. However, there is limited data on how often healthcare workers experience incivility specifically from patients, families, and visitors.

When defining and measuring incivility, several factors impede our ability to do so accurately. One factor is that incivility takes on so many different forms and much of it goes unreported by those who experience it. In addition, current tools may not always be able to distinguish incivility from other types of mistreatment, and overlap may be seen.

As Dr. Christine Porath states in her research on incivility, “identifying and studying incivility can be difficult, because bad behavior is often in the eye of the recipient“ [4]. In other words, our current research relies on the perceptions of the targets of incivility.

Furthermore, although incivility is a less severe form of mistreatment, there is often overlap among the different types of mistreatment. As current research in this area relies on survey responses, current tools may not always be able to distinguish between the subsets of mistreatment. With significant overlap between survey questions related to mistreatment, verbal abuse, racial and gender discrimination, and incivility, it can be challenging to determine an accurate prevalence. In addition, harmful behaviors may be labeled with varying terminology in the literature. Therefore, our review may not capture the full extent or impact of incivility.

In a study of workplace violence, for example, one category of violence included verbal abuse, which encompassed aggressive or inappropriate language (such as yelling), which made the worker feel threatened, scared, or uncomfortable. However, this study on workplace violence also included examples such as name-calling and rude language, which may be considered examples of incivility as well [5].

In a survey of more than 800 US physicians, close to 60% had heard offensive remarks about a personal characteristic in the previous five years. These included remarks about weight and youthfulness, as well as gender, race, and ethnicity, and thus encompassed incivility as well as gender and racially discriminatory language [6].

With these limitations of the current research kept in mind, multiple studies have documented incivility in healthcare and its associated impact. While there are different definitions, one of the early definitions of incivility in the literature comes from Anderson and Pearson, who defined it as “negative behaviors with low-intensity and unclear intention that damage the targeted person” [7]. Patel and Christman stated that “incivility is the perception of verbal or nonverbal actions that demean, dismiss or exclude an individual [8]. Examples of incivility can range from “belittling comments” to non-verbal gestures like eye-rolling or the rude use of a cell phone [9].

Dr. Porath has described incivility as rudeness, disrespect, or insensitive behavior. She specifies that it is subjective and includes various behaviors that run the gamut from “things like belittling someone or demeaning them in some way… offensive jokes… even things like not paying attention to someone.“ She specified that it is less intense in form than aggression or violence and may sometimes be a one-time episode [4].

Currently, instruments that fully measure incivility towards healthcare workers from patients and visitors are lacking. One tool that may be helpful as a starting point is the Nursing Incivility Scale. This scale includes eight subscales, one of which is incivility arising from patients and visitors, with the others surveying incivility from physicians, supervisors, other nurses, and general hospital staff [10]. Examples of items include “patients are condescending to me” and “patients criticize my job performance” [10]. The Incivility from Customers Scale has been used to measure negative behaviors in customer service industries, particularly retail industries [11]. Questions include “How often have customers blamed you for a problem you did not cause?” and “How often have customers made gestures (e.g., eye-rolling, sighing) to express their impatience?” [11]. While these tools may be helpful, a validated tool focused on incivility from patients, families, and visitors toward healthcare workers is currently not available.

Incivility can also be studied under the broader category of mistreatment. One validated scale for measuring mistreatment is the Mistreatment, Protection, and Respect (MPR) measure. This is a seven-item measure assessing participants’ experiences of different types of mistreatment in three categories: verbal mistreatment, sexual harassment, and physical violence [2]. Respondents are asked to identify the source of the mistreatment. In the category of verbal mistreatment, the measure refers to some behaviors that may be considered incivility. Specifically, the survey asks if the respondent has experienced a complaint or criticism “related to your professionalism (appearance or behavior),” or has experienced “verbal mistreatment such as name-calling or insulting jokes or humor.”

Risk factors for incivility

One literature review found that nurses in psychiatric inpatient units, emergency departments, pediatric departments, and neurosurgery departments experience the highest rates of incivility [12].

When looking at the characteristics of the healthcare worker that may predict the risk of experiencing incivility, there is a lack of robust literature. Data are lacking regarding factors such as gender, race, ethnicity, and age. Similarly, research is lacking as to factors such as job category and inpatient versus outpatient encounters. Research on more severe types of mistreatment has found that overall, ethnic and racial minority physicians report higher levels of discrimination and mistreatment from patients. Female physicians also report higher levels of discrimination and mistreatment, even after controlling for other factors [13].

In terms of patient characteristics, some research indicates that the emotional state of a patient greatly influences their likelihood to behave with incivility. Two emotions are most strongly associated with an increased incidence of incivility: anger and sadness [14]. Specifically, anger has been linked to an increased likelihood of “profanity, insults,... identity attacks, and threats” while sadness increases the likelihood of threats [14].

Consequences of incivility

Studies of rude behavior in various work environments have demonstrated that even mildly rude behavior may have a significant impact [15]. In healthcare, worker well-being as well as patient care may be impacted. As Credland and Whitfield discuss, even minor moments of incivility can accumulate and “erode staff morale, reduce confidence, and negatively impact well-being” of healthcare workers, all of which may contribute to burnout and intent to leave the profession [16]. Campana and Hammoud surveyed nurses and found that incivility from patients and their families was positively associated with burnout [17]. Clinical research coordinators were similarly studied by Mascaro et al. and were found to have significant rates of burnout due to incivility from patients and families [18]. Other studies have also documented a relationship between incivility and employee turnover intention [19].

In clinical settings, there have been multiple documented consequences for patient care. In one study, researchers found that incivility decreased nurses’ ability to provide patient-centered care and to communicate compassionately with patients [20]. Additional concerning findings in the incivility literature have come from randomized controlled trials (RCTs). In one trial, researchers randomly assigned NICU teams participating in a training workshop to two different conditions. The control condition included a neutral statement from the patient’s mother while in the other condition, the patient’s mother made rude statements (unrelated to the team’s performance). Exposure to rude statements resulted in adverse consequences, both in the diagnostic and intervention performance of the teams. Team processes were also negatively impacted, including information and workload sharing as well as helping and communication. Overall, researchers found that rudeness explained 39% of the variance in team-level general therapeutic outcomes [21]. Put in context, the experienced rudeness “explained more error than the levels of error that have been shown to result from sleep deprivation” [15].

In another RCT, 66% of nurses in the group exposed to incivility made a major error in cardiopulmonary resuscitation (CPR) performance [22]. Another study found that nurses were less likely to comply with hand hygiene protocols and had decreased rates of information sharing with other health professionals in the 24 hours following incidents of rudeness [23].

It is hypothesized that these effects occur because incidents of incivility divert cognitive resources away from the intervention being performed [21]. Another theory posits that the experience of incivility generates a negative affect that interferes with higher-order reasoning [24]. For example, one RCT utilized written clinical vignettes in which some patients were described as exhibiting neutral behaviors while others were described as exhibiting "difficult" behaviors. These included questioning their doctors' competence or accusing the doctor of discrimination. Mean diagnostic accuracy scores were significantly lower for vignettes involving "difficult" patients. The researchers concluded that doctors had to spend part of their mental resources on dealing with difficult patients' behaviors and this impeded their ability to adequately process clinical findings [24].

Qualitative interview studies have described some of the impacts on individual healthcare workers. In a small interview study of mental health professionals, it was found that client rudeness could have several potential negative impacts, including negative feelings about the professionals’ own competence in the role, as well as frustration toward clients and effects on their work [25]. A qualitative interview study of paramedics found that incivility was common, with all participants stating that they experienced incivility on at least a weekly basis from patients and their families as well as other professional groups [16]. They reported that this could lead them to question their actions and abilities, and could make decision-making more complex [16].

Prevention and response to incivility

Research on interventions that can be used to prevent incivility or ameliorate the negative effects is limited. In a review of workplace violence, a type of mistreatment that has received greater attention, Phillips wrote that “most studies on workplace violence have been designed to quantify the problem, and few have described research on experimental methods to prevent such violence [26]. He further writes that a review of the literature in 2000 had identified 137 studies on strategies to reduce workplace violence, but only nine had reported data on interventions, and all were described as reporting inconclusive results and using weak methods.

With this in mind, researchers have described some factors or approaches that may be protective or ameliorating in relation to incivility. For a summary of the interventions and approaches described here, please see Table 1.

**Table 1:** Prevention and Response to Incivility [1,21,25,27-28]

Category	Response Example
Prevention	The five-part intervention reduced incidents of incivility from 25 per 1000 to 9.5 per 1000. The intervention included a triage algorithm, an educational program to inform about the triage system, the use of TVs to broadcast waiting times, a professional mediator in the waiting room, and a video surveillance system
	Establish norms for patients, families, and visitors with a patient and visitor code of conduct
	Use signage that nudges respectful behavior and spells out unacceptable behavior
Response to incidents of incivility	Coaching frontline workers on de-escalation techniques
	Provide scripts for employees to use
	Create a clear chain of command
	Cognitive rehearsal training
	System-level interventions
Ameliorating the Effects of Incivility	Incorporate coaching techniques into medical training
	Leaders encourage and model recovery
	Respond naturally to rude behaviors as opposed to emotion work or acting
	Organizational level: provide organizational support and promote cultures of civility in the workplace
	Pre-incident training with cognitive bias modification training

Prevention

One program in a study by Touzet et al. was implemented department-wide and involved the creation of several new initiatives. This five-part intervention was successful in reducing incidents of incivility in the ophthalmic emergency department from 25 incidents per 1000 admitted patients to 9.5 incidents per 1000 [27]. Initiatives included a new triage algorithm used at intake to prioritize patients upon arrival, an educational program to introduce and inform patients about the triage system, and the use of TVs that broadcast waiting times. Additionally, a professional mediator circulated around the waiting room to intervene when patients became agitated, and a video surveillance system, connected to the security department, was used throughout the department [27].

Anecdotal reports also describe healthcare organizations taking action against patient-healthcare worker incivility. UMass Memorial Health, a healthcare network in Massachusetts, has worked to establish norms for patients, families, and visitors by introducing a patient and visitor code of conduct, with signs at entrances. At kiosks, visitors are asked to sign an agreement to adhere to the code of conduct [1]. Laura Flynn, Senior Director of Performance, Learning, and Education, stated that the goal was to create a respectful, safe environment. When the program was piloted, it noted that in just over a month, it had more than 56,000 signed agreements for the code of conduct and only four visitors were asked to leave during that time.

The use of nudges has also proven useful. Nudges are short, personalized recommendations with a clear call to action [1]. UMass Memorial Health displays signage to nudge patients and visitors to exhibit respectful behavior. The welcome sign says “help us keep this a safe space of healing, kindness, and respect.” In smaller font at the bottom are the hospital’s standards of respect. In hospital units and the emergency department, signs spell out unacceptable behavior, specifically including yelling, swearing, spitting, and making offensive remarks about race, ethnicity, or religion [1].

Responding to Incivility Incidents

Despite robust prevention efforts, it is recognized that incivility will continue to occur. Therefore, another avenue of research has focused on best practices for responding to patient incivility.

One recommended technique is coaching frontline workers on de-escalation techniques. UMass Memorial has established a template that provides scripts for employees to use to respond to incivility. Furthermore, if efforts are not successful, there is a clear chain of command to ask for help. For example, workers may first get their manager involved, and if needed, can also contact the patient advocacy staff for guidance [1].

Other potential approaches to patient incivility may be found in the literature relating to incivility between other groups. For example, one study on nurse-to-nurse incivility tested an intervention aimed at helping nurses identify incivility and learn how to respond to it through cognitive rehearsal training [28]. This intervention proved effective at helping nurses recognize incivility and feel more equipped to respond to it while also reducing the perceived incidents of incivility over time [28].

The literature on mistreatment also offers some guidance. The AMA Code of Medical Ethics recommends transferring the care of the patient to another physician if the patient refuses to “modify their conduct” [29]. One study on the mistreatment of physicians recommends that hospitals and healthcare institutions can protect the occupational health of their practitioners by implementing interventions on “implicit bias training, leadership development, anonymous reporting systems, and bystander trainings, among others,” which have been shown to have protective effects on well-being [2].

Ameliorating the Effects of Incivility

There is limited research on best practices for recovery after incivility. There are some suggestions that incorporating coping techniques into medical training may help healthcare professionals cope with and respond to incivility while reducing its negative effects [25]. For leaders, experts recommend encouraging and modeling types of recovery practices [1].

At the individual level, it was found that nurses who respond to incivility using emotion work or "acting" experience more fatigue than nurses who respond naturally and with their true emotions [30]. Emotion work is when one attempts to modulate or change their emotional response in the face of negative experiences.

At the organizational level, one study found that work environments that provide more organizational support as well as support from work colleagues that resembles family support help buffer against the negative effects of incivility [31]. The importance of organizational and workplace support is echoed in another study that found that workplace cultures of civility, with social support from coworkers, provided a buffering effect that helped protect against the “spiraling” negative consequences of everyday experiences of workplace incivility [32].

One RCT was able to demonstrate the benefits of pre-incident training. In this study, two NICU teams participating in a training workshop were randomly assigned to experience rude behavior. One group received a preventative intervention (before the rudeness) and the other group received a therapeutic intervention (after the rudeness). In the preventive intervention, the team underwent cognitive bias modification training prior to the rude incident. This consisted of a 20-minute “computer game“ and targeted negative interpretations of emotional displays. This training mitigated most of the adverse effects of rudeness, with effects sustained throughout the day [21]. In contrast, the post-incident intervention, consisting of a narrative exercise, had no significant effect.

In terms of occupational cultural beliefs around incivility, it is unknown how these may impact a worker’s response to rude behavior from patients. Studies have found that many healthcare professionals view incivility from patients as an expected part of the career and process of providing healthcare [33]. This sentiment was also echoed in a study of mental health professionals who reported consistently that rudeness was “part of the job” no matter the therapeutic relationship [25]. It is believed that this belief may have positive or negative ramifications. This acceptance could serve as a buffer to prevent harm resulting from incivility, but it could also reduce the likelihood of challenging or preventing incivility from patients in the future [25].

## Conclusions

As frontline workers face increasing incivility from the populations that they serve, it is important to recognize the resulting consequences. Also of urgent importance is the need to determine best practices in prevention and response to these incidents, with a focus on protecting healthcare workers and ensuring optimal patient care. Mistreatment covers a spectrum of negative behaviors, and although rudeness and incivility are not as severe as other types of mistreatment, they may still result in significant impacts on healthcare worker well-being and patient care. Consequences include negative effects on well-being, increased rates of burnout, and increased intent to leave the profession. Randomized controlled trials have demonstrated that rude behavior can also adversely affect the diagnostic and intervention performance of clinical care teams, as well as negatively impacting team processes. Preventing and responding to incivility requires a multi-pronged approach. Although research on interventions that may prevent or ameliorate the negative consequences of incivility is currently limited, there are some promising interventions that have been described in the literature. From the individual to the organizational level, effective strategies will need to be developed, studied, and implemented, in order to prevent the negative consequences of these increasingly common behaviors in the workplace.
